# Suppression of the interleukin-1β-induced inflammatory response of human Chang liver cells by acute and subacute exposure to alcohol: an *in vitro* study

**DOI:** 10.3325/cmj.2018.59.46

**Published:** 2018-04

**Authors:** Katharina Mörs, Shinwan Kany, Jason-Alexander Hörauf, Nils Wagner, Claudia Neunaber, Mario Perl, Ingo Marzi, Borna Relja

**Affiliations:** 1 Department of Trauma, Hand and Reconstructive Surgery, Goethe-University, Frankfurt am Main, Germany; 2 Department of Trauma Surgery, Medical School Hannover, Hannover, Germany; 3 Department of Trauma Surgery, Trauma Center Murnau, Murnau, Germany

## Abstract

**Aim:**

To evaluate protective immunosuppressive dose and time-dependent effects of ethanol in an *in vitro* model of acute inflammation in human Chang liver cells.

**Method:**

The study was performed in 2016 and 2017 in the research laboratory of the Department of Trauma, Hand and Reconstructive Surgery, the University Hospital of the Goethe-University Frankfurt. Chang liver cells were stimulated with either interleukin (IL)-1β or IL-6 and subsequently treated with low-dose ethanol (85 mmol/L) or high-dose ethanol (170 mmol/L) for one hour (acute exposure) or 72 hours (subacute exposure). IL-6 and IL-1β release were determined by enzyme-linked immunosorbent assay. Neutrophil adhesion to Chang liver monolayers, production of reactive oxygen species, and apoptosis or necrosis were analyzed.

**Results:**

Contrary to high-dose ethanol, acute low-dose ethanol exposure significantly reduced IL-1β-induced IL-6 and IL-6-induced IL-1β release (*P* < 0.05). Subacute ethanol exposure did not change proinflammatory cytokine release. Acute low-dose ethanol exposure significantly decreased inflammation-induced formation of reactive oxygen species (*P* < 0.05) and significantly improved cell survival (*P* < 0.05). Neither acute nor subacute high-dose ethanol exposure significantly changed inflammation-induced changes in reactive oxygen species or survival. Acute and subacute ethanol exposure, independently of the dose, significantly decreased neutrophil adhesion to inflamed Chang liver cells (*P* < 0.05).

**Conclusion:**

Acute treatment of inflamed Chang liver cells with ethanol showed its immunosuppressive potential. However, the observed effects were limited to low-dose setting, indicating the relevance of ethanol dose in the modulation of inflammatory cell response.

Chronic or excessive alcohol misuse is associated with several pathogenic mechanisms that structurally alter hepatic epithelial cells, leading to alcoholic steatohepatosis or alcoholic liver disease, with tissue remodeling and damage (1-3). Alcohol misuse history induces and aggravates the inflammatory cascade, with massive generation and release of proinflammatory cytokines, activation of resident macrophages, and oxidative stress, thereby promoting cellular damage (4-8). Alcoholic hepatitis was associated with elevated systemic levels of proinflammatory interleukin (IL)-8, which are closely linked to neutrophil chemotaxis and hepatic neutrophil infiltration, and correlate with the severity of alcoholic liver disease (9-12). Similarly, chronic alcohol exposure increased the hepatic and systemic levels of proinflammatory IL-6 and tumor necrosis factor (TNF) α (13), and the levels of reactive oxygen species (ROS) (6,14,15). Additionally, rats fed chronically with an alcohol-containing diet had lower hepatocyte viability and increased apoptosis rates (16,17). These processes end up in serious pathologies apart from alcoholic liver disease, such as hypertension, diabetes, or cancer (18), but are also associated with the development of clinical infections (19).

In contrast, critically ill non-trauma patients had significantly decreased values of circulating neutrophils after acute alcohol misuse in comparison with chronic misuse (20). *In vitro* studies showed that acute alcohol exposure diminished the release of proinflammatory cytokines, eg, IL-8 and interaction of neutrophils with endothelial and epithelial cells (21,22). These effects were confirmed *in vivo*, showing lowered local hepatic and systemic IL-6 levels and neutrophil infiltration in the liver upon acute exposure to alcohol (23). Also, acute alcohol exposure was shown *in vitro* to impair neutrophilic capability for ROS production (24). Taken together, experimental and epidemiological data confirm “positive” effects of moderate alcohol exposure to be associated with a reduced risk of cardiovascular disease events, diabetes, or coagulopathy (25,26).

In summary, while chronic alcohol misuse was associated with negative clinical outcomes, acute alcohol misuse had no deleterious influence on post-injury outcome, or even exerted “positive” effects on the clinical course (27-30). However, it is not evident which dose or duration of alcohol exposure exerts “positive” anti-inflammatory effects. Interestingly, a short-term exposure of isolated neutrophils to different alcohol doses has shown a dose-dependent inhibitory effect on oxidative stress and cytokine production (IL-8 and TNFα) (31).

Therefore, we postulated a dose- and time-dependent immune-suppressive potential of alcohol. We also postulated that exposing Chang liver cells to low-dose of alcohol would reduce the release of proinflammatory cytokines IL-6 and IL-1β, adhesion capability of neutrophils, and ROS formation, and increase viability in this *in vitro* model of epithelial inflammation.

## Material and methods

This experimental *in vitro* study was performed in 2016 and 2017 in the research laboratory of the Department of Trauma, Hand and Reconstructive Surgery, the University Hospital of the Goethe-University Frankfurt, with commercially available human Chang liver cells (Cell Line Services, Heidelberg, Germany), which is why no ethical approval was required.

### Cell culture

Chang liver cell line with epithelial morphology and a subline of HeLa cells were cultured at 37°C under 5% carbon dioxide (CO_2_) in Roswell Park Memorial Institute (RPMI)-1640 medium (Seromed, Berlin, Germany) supplemented with 10% heat-inactivated fetal calf serum, 100 IU/mL penicillin, 100 µg/mL streptomycin (Gibco, Karlsruhe, Germany), and 20 mmol/L 4-(2-hydroxyethyl)-1-piperazineethanesulfonic acid buffer (Sigma, Steinheim, Germany). Culture media were replaced every second or third day. Peripheral blood polymorphonuclear neutrophils (PMN) were isolated by density-gradient centrifugation (Polymorphprep, Nycomed, Oslo, Norway) according to the manufacturer’s instructions. After isolation, PMN were cultured in RPMI-1640 medium, and their number and viability were determined with trypan blue exclusion assay. Only cell cultures with a purity of >95% were used.

### Cell stimulation

To analyze the time- and dose-dependent release of proinflammatory cytokines by Chang liver cells, IL-1β and IL-6 release after acute or subacute exposure to ethanol was determined. The concentrations of IL-1β, IL-6, and ethanol were chosen on the basis of previous studies to allow for better data comparison (22,28,31-33). Chang liver cells were stimulated with either recombinant human IL-1β or IL-6 (1 ng/mL and 10 ng/mL, respectively, R&D Systems, Wiesbaden, Germany) for 24 hours. Afterwards, without replacing the medium, ethanol was added, and the effects of acute and subacute ethanol exposure were evaluated after one hour and 72 hours, respectively. To evaluate the dose-dependent responses, ethanol was used at the low-dose of 85 mmol/L or high-dose of 170 mmol/L, as described previously (28,32,34). A total of 16 experimental runs was performed.

### Apoptosis and cell viability measurement

Chang liver cells were treated as described above and incubated with propidium iodide (PI) and annexin V-conjugated fluorescein isothiocyanate (FITC) from the annexin V-FITC Apoptosis Detection Kit 1 (Becton Dickinson, Heidelberg, Germany) according to the manufacturer’s instructions. Annexin V-FITC/PI binding was evaluated by flow cytometry, using a BD FACS Canto 2 and FACD DIVA^TM^ software (Becton Dickinson). The population of PI-negative/annexin V-negative cells constituted vital cells, and data were expressed as percentage of vital cells referred to all measured cells.

The viability of neutrophils was assessed by trypan blue exclusion assay. Isolated neutrophils were stained with 0.4% trypan blue, and about 100 cells were counted after each isolation. The mean percentage of viability was >99%. This experiment was repeated four times.

### Quantification of cytokine production

To determine the effects of ethanol on the cytokine production, Chang liver cells were pre-incubated with low-dose or high-dose ethanol for one or 72 hours after stimulation with IL-1β or IL-6. Then, IL-6 and IL-1β were measured in culture supernatants using IL-6/IL-1β enzyme-linked immunosorbent assay (ELISA) sets (Diaclone, Besançon, France) according to the manufacturer’s instructions. ELISA was performed using Infinite M200 microplate reader (Tecan, Männedorf, Switzerland). This experiment was repeated three times.

### Monolayer adhesion assay

To analyze PMN adhesion to pre-treated cells, Chang liver cells were transferred into 24-well multiplates (Sarsted, Nümbrecht, Germany) in a complete RPMI-1640 medium. When a confluence of ~ 80% was reached, the cells were stimulated with IL-1β for 24 hours and treated with low-dose or high-dose ethanol for one hour (acute exposure) or 72 hours (subacute exposure). Freshly isolated PMNs were counted and adjusted to 5 × 10^4^ vital cells/well and then carefully added to the Chang liver monolayers. After incubation for 15 minutes at 37°C under 5% CO_2_, non-adherent PMNs were washed off three times using pre-warmed (37°C) complete RPMI-1640 medium. The remaining PMNs were fixed using 1% glutaraldehyde. Adherent PMN were counted in five different fields of defined size (5 × 0.25 mm^2^) using a phase contrast microscope (×10 objective), and the mean cellular adhesion rate was calculated as the ratio to unstimulated controls (%). The assay was performed as described previously (22). This experiment was repeated five times.

### Oxidative burst analysis

Chang liver cells were cultured at 37°C under 5% CO_2_ and treated as described above. The cells were detached from multiplate-wells by using accutase, transferred into polystyrene FACS tubes (BD Pharmingen, Heidelberg, Germany), and washed with RPMI-1640 medium at 400 g for five minutes. Thereafter, the cells were resuspended in a 100-μL culture medium with supplements, and 20 μL of CM-H2DCFDA (General Oxidative Stress Indicator Kit, Invitrogen, Darmstadt, Germany) was added to each sample, as suggested by the manufacturer. The samples were then incubated for 30 minutes at 37°C under 5% CO_2,_ and the supernatant was discarded. Then, 400 μL of cell culture medium with supplements was added to each sample. After 60 minutes at 37°C under 5% CO_2_, the cells were washed with 4 mL phosphate buffered saline supplemented with 0.5% bovine serum albumin (FACS buffer) and centrifuged at 400 g for 5 minutes. The supernatant was removed, and the cells were diluted in 200-µL FACS buffer and subjected to flow cytometry using BD FACS Canto 2 and FACD DIVA^TM^ software. The Chang liver cells were gated by the corresponding forward- and side-scatter scan. From each sample, a minimum of 20 000 cells was measured. The percentage of cells positive for oxidative stress was calculated relative to the whole cell population of unstained cells. This experiment was repeated four times.

### Statistical analysis

The normality of data distribution was tested using Kolmogorov-Smirnov test. Since data were not normally distributed, Kruskal-Wallis with Dunn’s multiple corrections test was applied to test the differences between the groups. Data were presented as median and interquartile range. Statistical analysis was performed using GraphPad Prism 6 (GraphPad Software Inc., San Diego, CA, USA; the license holder is the corresponding author). *P*<0.05 was considered significant.

## Results

### Cytokine release after proinflammatory stimulation and subsequent ethanol exposure of Chang liver cells

**Figure 1:**
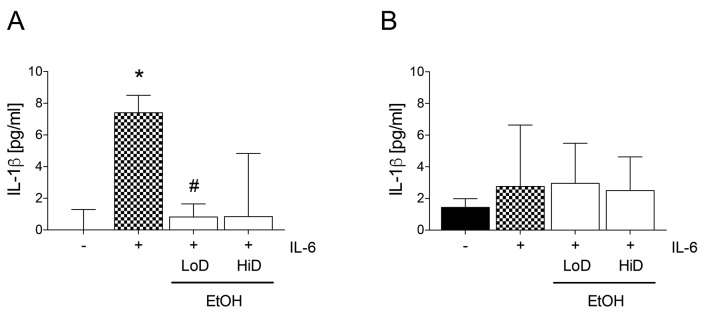
Effects of acute and subacute exposure to low-dose and high-dose ethanol (EtOH) on interleukin-1β release after interleukin-6 stimulation. After the stimulation with interleukin (IL)-6 (10 ng/mL) for 24 hours, Chang liver cells were exposed to EtOH (low-dose, LoD = 85 mmol/L, high-dose, HiD = 170 mmol/L) for one hour (acute, **A**) or 72 hours (subacute, **B**). After the incubation periods, supernatants were analyzed for IL-1β concentrations. The data are presented as median and interquartile range. Black bar: control cells; checkered bar: cells stimulated with IL-6; empty bar: cells stimulated with IL-6 and exposed to EtOH. *P < 0.05 vs not pretreated and not stimulated cells; ^#^*P* < 0.05 vs not pretreated but stimulated control.

Stimulation with IL-6 significantly increased the IL-1β release (*P* < 0.05, Figure 1). Both low-dose and high-dose acute ethanol exposure further significantly decreased the IL-1β release (*P* < 0.05, Figure 1A) in comparison with stimulated controls. The decreasing effect was more pronounced in low-dose than in high-dose setting, but the difference was not significant. Subacute ethanol exposure significantly increased IL-1β release after IL-6 stimulation in comparison with unstimulated controls (*P* < 0.05, Figure 1B). After both low-dose and high-dose ethanol subacute exposure, IL-1β release showed an increasing trend; but the increase was not significant (Figure 1B).

**Figure 2:**
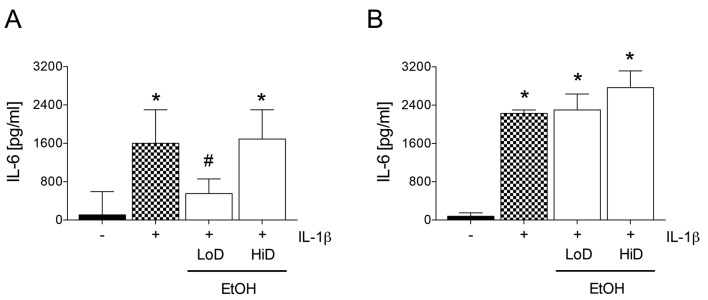
Effects of acute and subacute exposure to low-dose and high-dose ethanol (EtOH) on interleukin-6 release after interleukin-1β stimulation. After the stimulation with interleukin (IL)-1β (10 ng/mL) for 24 hours, Chang liver cells were exposed to EtOH (low-dose, LoD = 85 mmol/L, high-dose, HiD = 170 mmol/L) for one hour (acute, **A**) or 72 hours (subacute, **B**). After the incubation periods, supernatants were analyzed for IL-6 concentrations. The data are presented as median and interquartile range. Black bar: control cells; checkered bar: cells stimulated with IL-1β; empty bar: cells stimulated with IL-1β and exposed to EtOH. *P < 0.05 vs not pretreated and not stimulated cells; ^#^*P* < 0.05 vs not pretreated but stimulated control.

Stimulation with IL-1β significantly increased IL-6 release (*P* < 0.05, Figure 2). Subsequent low-dose acute ethanol exposure significantly decreased the IL-6 release in comparison with stimulated controls (*P* < 0.05, Figure 2A). High-dose acute ethanol exposure had no significant effect on IL-6 release (Figure 2A). Either low-dose or high-dose subacute ethanol exposure did not change the significantly increased IL-6 release after IL-1β stimulation (Figure 2B).

### Adherence of polymorphonuclear leukocytes after ethanol exposure and interleukin-1β stimulation

**Figure 3:**
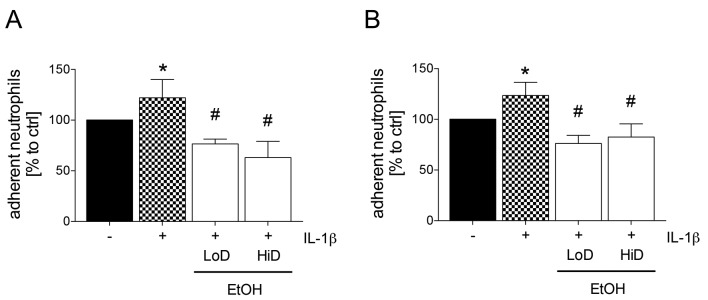
Effects of acute and subacute exposure to low-dose and high-dose ethanol (EtOH) on the adhesiveness of neutrophils to Chang liver cells after interleukin-1β stimulation. After the stimulation with IL-1β (1 ng/mL) for 24 hours, Chang liver cells were exposed to EtOH (low-dose, LoD = 85 mmol/L, high-dose, HiD = 170 mmol/L) for one hour (acute, **A**) or 72 hours (subacute, **B**). After the incubation periods, isolated human neutrophils were added and the adhesion capacity was determined. The mean adhesion rates are presented as median and interquartile range. Black bar: control cells; checkered bar: cells stimulated with IL-1β; empty bar: cells stimulated with IL-1β and exposed to EtOH. *P < 0.05 vs not pretreated and not stimulated cells; ^#^*P* < 0.05 vs not pretreated but stimulated control.

Stimulation with IL-1β significantly increased the adhesion capacity of PMNs to Chang liver monolayers in comparison with unstimulated controls (*P* < 0.05, Figure 3). After IL-1β stimulation and low-dose acute ethanol exposure and further high-dose acute ethanol exposure, adhesion capacity of PMNs was significantly decreased in comparison with controls (*P* < 0.05, Figure 3A). Both low-dose and high-dose subacute ethanol exposure significantly decreased adhesion capacity of PMNs, but there was no significant difference between the ethanol concentrations (*P* < 0.05, Figure 3B).

### ROS production upon acute and subacute exposure to ethanol after interleukin-1β stimulation

**Figure 4:**
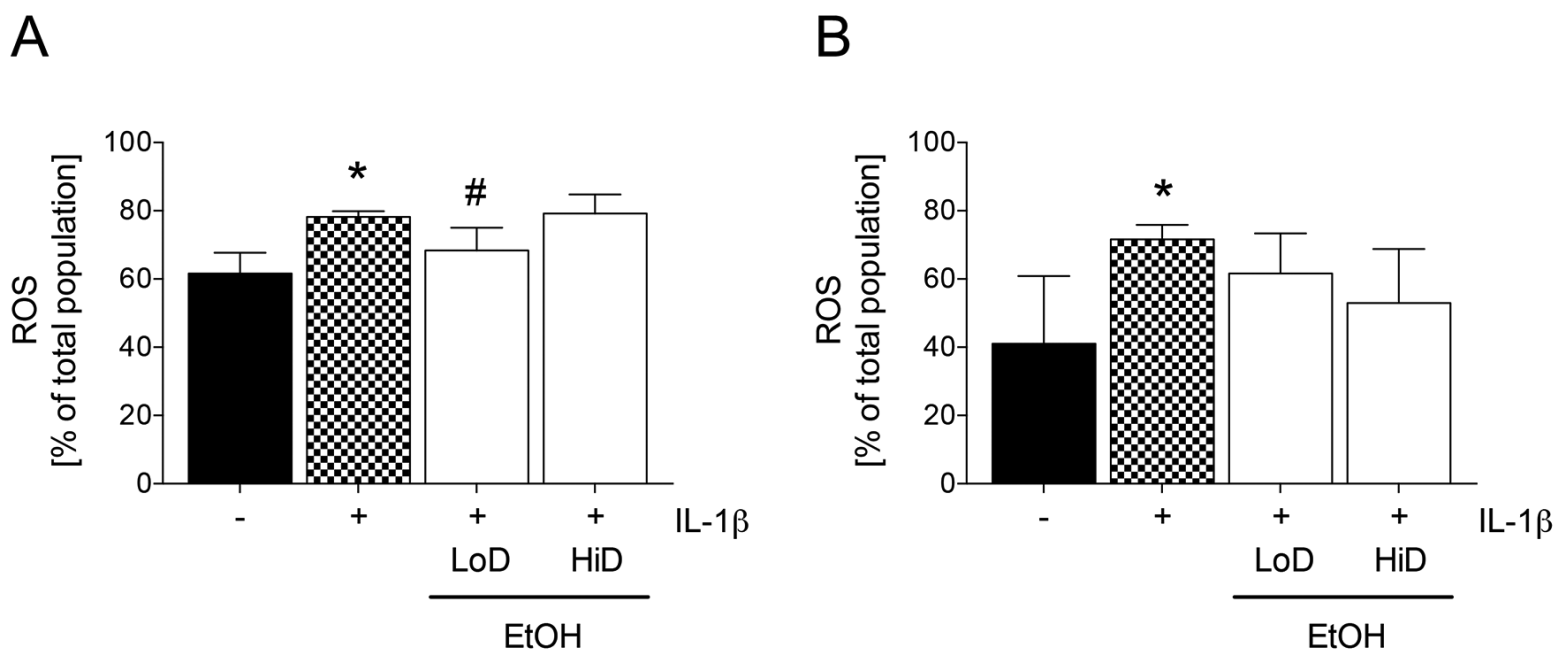
Effects of acute and subacute exposure to low-dose and high-dose ethanol (EtOH) on production of reactive oxygen species (ROS) in Chang liver cells after interleukin-1β stimulation. After the stimulation with interleukin-1β (1 ng/mL) for 24 hours, Chang liver cells were exposed to EtOH (low-dose, LoD = 85 mmol/L, high-dose, HiD = 170 mmol/L) for one hour (acute, **A**) or 72 hours (subacute, **B**). After the incubation periods, the production of ROS was determined. The percentages of ROS-positive cells from total measured population are presented as median and interquartile range. Black bar: control cells; checkered bar: cells stimulated with IL-1β; empty bar: cells stimulated with IL-1β and exposed to EtOH. *P < 0.05 vs not pretreated and not stimulated cells; ^#^*P* < 0.05 vs not pretreated but stimulated control.

Stimulation with IL-1β significantly increased ROS production compared with unstimulated controls (*P* < 0.05, Figure 4). Low-dose acute ethanol exposure, significantly decreased IL-1β-induced ROS production (*P* < 0.05), while high-dose acute ethanol exposure did not change it (Figure 4A). Subacute ethanol exposure did not significantly change IL-1β induced-ROS production (Figure 4B).

### Apoptose rates after acute and subacute exposure to ethanol after interleukin-1β stimulation

**Figure 5:**
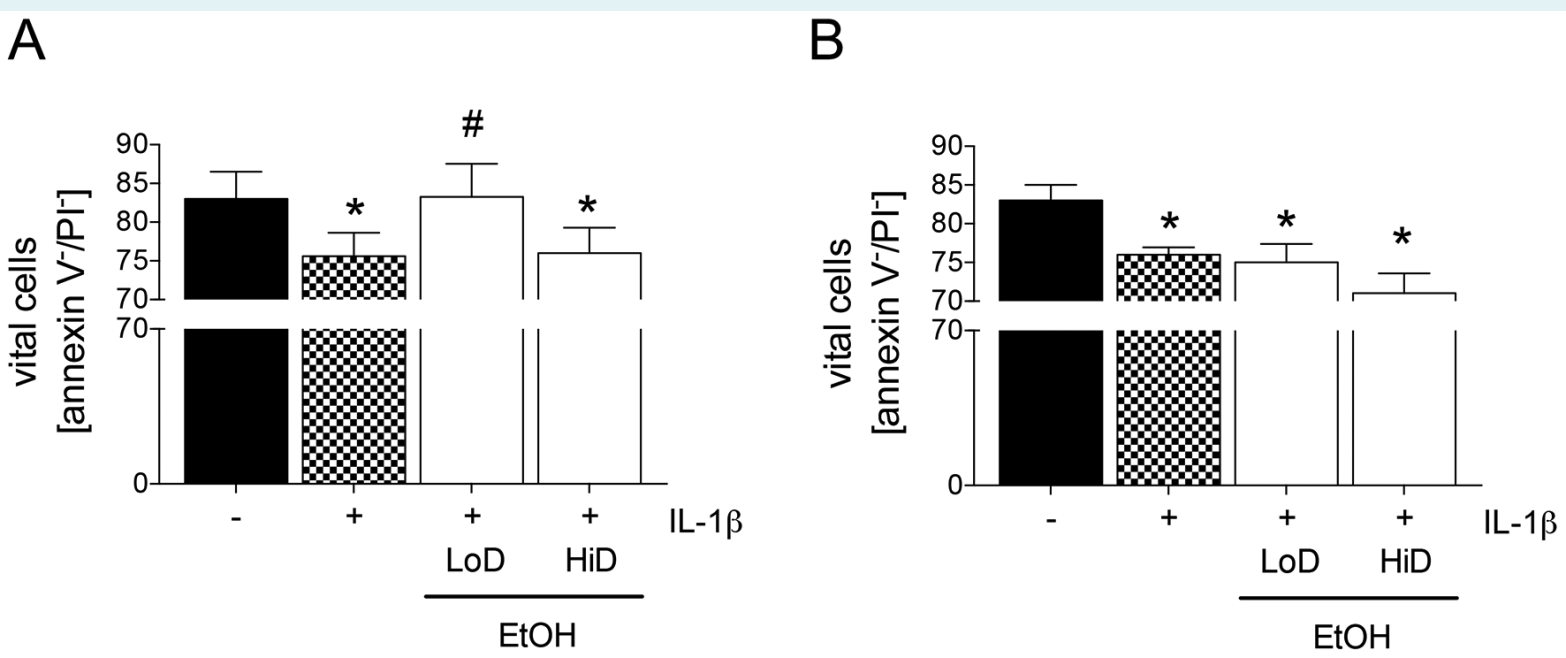
Effects of acute and subacute exposure to low-dose and high-dose ethanol (EtOH) on cell viability/apoptosis of Chang liver cells after interleukin-1β stimulation. After the stimulation with IL-1β (1 ng/mL) for 24 hours, Chang liver cells were exposed to EtOH (low-dose, LoD = 85 mmol/L, high-dose, HiD = 170 mmol/L) for one hour (acute, **A**) or 72 hours (subacute, **B**). After the incubation periods, the percentage of vital cells (propidium iodide [PI], PI-negative, and annexin V-negative cells) referred to all measured cells was determined. Data are presented as median and interquartile range. Black bar: control cells; checkered bar: cells stimulated with IL-1β; empty bar: cells stimulated with IL-1β and exposed to EtOH. *P < 0.05 vs not pretreated and not stimulated cells; ^#^*P* < 0.05 vs not pretreated but stimulated control, ctrl.

With higher concentration and higher duration of exposure, ethanol caused a certain loss of the pre-formed confluent monolayer and partly of its normal shape. Stimulation with IL-1β significantly decreased the survival rate of Chang liver cells in comparison with unstimulated controls (*P* < 0.05, Figure 5). Low-dose acute ethanol exposure significantly increased IL-1β-reduced cell survival (*P* < 0.05), while high-dose acute ethanol exposure did not change it in comparison with stimulated controls (Figure 5A). Subacute ethanol exposure did not significantly change the IL-1β-reduced cell survival (Figure 5B). High-dose subacute ethanol exposure suppressed the cell survival compared to IL-β stimulated controls; however, this difference was not significant.

## Discussion

We showed that immunosuppressive properties of ethanol in the setting of acute cellular inflammation were dose and time dependent. The observed anti-inflammatory effects of alcohol on inflamed human Chang liver cells *in vitro* were mainly limited to low-dose acute ethanol administration.

Low-dose acute ethanol exposure of Chang liver cells significantly decreased the proinflammatory cytokine release after stimulation with either IL-1β or IL-6. Moreover, this immunosuppressive effect was reflected by alcohol-reduced adherence rates of isolated neutrophils to stimulated Chang liver cells. While subacute ethanol exposure did not alter the secretory capacity, both low- and high-dose subacute ethanol exposure suppressed neutrophil adhesion. Acute ethanol exposure decreased ROS formation in a dose-dependent manner, which was not observed under subacute exposure conditions. In parallel, the ROS formation reduced by acute low-dose ethanol exposure was associated with increased cellular viability and less inflammation-induced apoptosis or necrosis. Contrary to this, high-dose acute ethanol exposure did not induce these effects. In contrast to subacute ethanol exposure, acute exposure indicated the immunosuppressive potential of ethanol. Nonetheless, the observed anti-inflammatory effects of ethanol were mainly observed in the low-dose setting, indicating the relevance of ethanol dose rather than the duration of exposure.

The observed immunosuppressive effects are in line with previous studies reporting anti-inflammatory potential of acute or moderate alcohol exposure in several models of inflammation (21,22,24,33). Nevertheless, there are serious inconsistencies regarding alcohol’s effects on the inflammatory response. While numerous findings indicate deleterious effects of chronic or excessive alcohol consumption, its use in moderate dose or acute settings has been linked to “positive” effects (23,25,27,28,35,36). Harmful effects of chronic misuse, ie, increased production and release of proinflammatory cytokines and increased tissue infiltration with neutrophils have been well-described as important factors in the pathogenesis of organ injury (37,38). In contrast, a decreased IL-6 production of murine macrophages was observed early after a single dose of alcohol (39). Similarly, IL-6 release was reduced in a time and dose dependent manner after acute alcohol exposure of macrophages and their stimulation with lipopolysaccharide (LPS) (7). Similar data were shown in an animal model of hemorrhagic shock, where acute alcohol exposure before hemorrhage significantly suppressed liver and systemic inflammatory responses, including IL-6 levels, and improved survival rates in the intoxicated group after hemorrhage (23). Furthermore, comparable to our data, MacGregor et al (40) have shown that short-term alcohol exposure decreased the proinflammatory cytokine levels and the neutrophil adhesion to endothelial and epithelial cells. The decreased neutrophil adhesion to Chang liver cells observed in our study after acute and subacute alcohol exposure is in line with Jonsson and Palmblad`s study (41), who found alcohol-diminished LPS-induced neutrophil adhesion to human umbilical vein endothelial cells, which they linked to the reduced activation of NF-kappaB. Our group previously found similar effects of acute alcohol exposure on inflammation-induced neutrophil adhesion capacity to lung epithelial cells (22). These and other data clearly indicate possible “positive” effects of acute alcohol-induced immune-suppression in models of acute inflammation.

However, in several burn-models, additional alcohol administration has shown adverse effects. As such, increased neutrophil infiltration, edema formation in lung tissue, and potentiated LPS-induced activation of Kupffer cells was observed upon oral alcohol gavage and burn injury (42,43). Systemic and adipose tissue IL-6 levels were elevated in mice undergoing single binge alcohol exposure followed by burn, while even more pronounced cytokine response was induced by episodic alcohol exposure followed by burn (44). These findings are contradictory to our data. However, they underline the influence of differing regimes and models on alcohol effects, which clearly depend on the exposure timeline and alcohol dosage.

ROS are required for microbicidal killing but may also amplify the local inflammation and subsequent cellular damage (45). This is also reflected by fewer ROS and higher rates of vital cells after acute low-dose alcohol exposure in our study. In line with these findings, reduced ROS formation and the associated reduced cell death/apoptosis were reported after acute alcohol exposure in an *in vivo* model of acute inflammation (36). However, there are also contradictory results, showing increased ROS in hepatocytes after acute alcohol-treatment (16). The differences may be explained by the applied dose, which was considerably lower (1 and 10 mmol/L ethanol) than in our study. Regarding cell death, there are both, conflictive and supporting, studies to our data (46-49). Therefore, acute alcohol use may reduce apoptosis/cell death in models of acute cell stress, eg, ischemia, while it may have negative effects on cell viability in models without acute stress (46-49). In our study, the observed effects of acute alcohol exposure may be considered “positive,” since it reduced inflammation-induced cell death and ROS formation. On the other hand, alcohol`s high dose but also subacute exposure did not influence inflammation-induced ROS formation and cell death. Considering these and other data, there seems to be a narrow range of alcohol doses and exposure durations that exert beneficial effects in experimental models.

One of the limitations of our study is that the used cell line with the epithelial morphology is actually a subline of HeLa cells, which was isolated from the liver. Another limitation is that only two doses of ethanol and only two time points of exposure were used. Further studies should investigate prolonged exposure to alcohol with different incubation periods in order to understand mechanistically the dose- and time-dependent development of pathologies, such as chronic organ diseases or even cancer in different cell entities. The small sample size certainly limits the statistical power of the study. The study design and the sample size are based on previous *in vitro* studies from our group (22,50), and the observed significant results upon stimulation with cytokines as compared to unstimulated controls in the present study. Additionally, other relevant pro- and anti-inflammatory cytokines should be included in further analyses. In spite of these limitations, this study provides important insights into the dose- and time-dependency of ethanol’s mode of action in acute inflammatory setting.

## References

[1] Cubero FJ, Urtasun R, Nieto N (2009). Alcohol and liver fibrosis.. Semin Liver Dis.

[2] Nagata K, Suzuki H, Sakaguchi S (2007). Common pathogenic mechanism in development progression of liver injury caused by non-alcoholic or alcoholic steatohepatitis.. J Toxicol Sci.

[3] O’Shea RS, Dasarathy S, McCullough AJ (2010). Practice Guideline Committee of the American Association for the Study of Liver Diseases; Practice Parameters Committee of the American College of Gastroenterology. Alcoholic liver disease.. Hepatology.

[4] Crews FT, Bechara R, Brown LA, Guidot DM, Mandrekar P, Oak S (2006). Cytokines and alcohol.. Alcohol Clin Exp Res.

[5] Jaeschke H (2002). Neutrophil-mediated tissue injury in alcoholic hepatitis.. Alcohol.

[6] Jaeschke H (2011). Reactive oxygen and mechanisms of inflammatory liver injury: Present concepts.. J Gastroenterol Hepatol.

[7] Karavitis J, Kovacs EJ (2011). Macrophage phagocytosis: effects of environmental pollutants, alcohol, cigarette smoke, and other external factors.. J Leukoc Biol.

[8] Szabo G, Mandrekar P (2009). A recent perspective on alcohol, immunity, and host defense.. Alcohol Clin Exp Res.

[9] Bautista AP (2002). Neutrophilic infiltration in alcoholic hepatitis.. Alcohol.

[10] Hill DB, Marsano LS, McClain CJ (1993). Increased plasma interleukin-8 concentrations in alcoholic hepatitis.. Hepatology.

[11] Huang YS, Chan CY, Wu JC, Pai CH, Chao Y, Lee SD (1996). Serum levels of interleukin-8 in alcoholic liver disease: relationship with disease stage, biochemical parameters and survival.. J Hepatol.

[12] Sheron N, Bird G, Koskinas J, Portmann B, Ceska M, Lindley I (1993). Circulating and tissue levels of the neutrophil chemotaxin interleukin-8 are elevated in severe acute alcoholic hepatitis, and tissue levels correlate with neutrophil infiltration.. Hepatology.

[13] Maraslioglu M, Oppermann E, Blattner C, Weber R, Henrich D, Jobin C (2014). Chronic ethanol feeding modulates inflammatory mediators, activation of nuclear factor-kappaB, and responsiveness to endotoxin in murine Kupffer cells and circulating leukocytes.. Mediators Inflamm.

[14] Mittal M, Siddiqui MR, Tran K, Reddy SP, Malik AB (2014). Reactive oxygen species in inflammation and tissue injury.. Antioxid Redox Signal.

[15] Zima T, Fialova L, Mestek O, Janebova M, Crkovska J, Malbohan I (2001). Oxidative stress, metabolism of ethanol and alcohol-related diseases.. J Biomed Sci.

[16] Bailey SM, Cunningham CC (1998). Acute and chronic ethanol increases reactive oxygen species generation and decreases viability in fresh, isolated rat hepatocytes.. Hepatology.

[17] Baroni GS, Marucci L, Benedetti A, Mancini R, Jezequel AM, Orlandi F (1994). Chronic ethanol feeding increases apoptosis and cell proliferation in rat liver.. J Hepatol.

[18] Ahmed FE (1995). Toxicological effects of ethanol on human health.. Crit Rev Toxicol.

[19] de Roux A, Cavalcanti M, Marcos MA, Garcia E, Ewig S, Mensa J (2006). Impact of alcohol abuse in the etiology and severity of community-acquired pneumonia.. Chest.

[20] Gacouin A, Roussel M, Le Priol J, Azzaoui I, Uhel F, Fest T (2014). Acute alcohol exposure has an independent impact on C-reactive protein levels, neutrophil CD64 expression, and subsets of circulating white blood cells differentiated by flow cytometry in nontrauma patients.. Shock.

[21] Boe DM, Nelson S, Zhang P, Bagby GJ (2001). Acute ethanol intoxication suppresses lung chemokine production following infection with Streptococcus pneumoniae.. J Infect Dis.

[22] Relja B, Omid N, Schaible A, Perl M, Meier S, Oppermann E (2015). Pre- or post-treatment with ethanol and ethyl pyruvate results in distinct anti-inflammatory responses of human lung epithelial cells triggered by interleukin-6.. Mol Med Rep.

[23] Relja B, Hohn C, Bormann F, Seyboth K, Henrich D, Marzi I (2012). Acute alcohol intoxication reduces mortality, inflammatory responses and hepatic injury after haemorrhage and resuscitation in vivo.. Br J Pharmacol.

[24] Patel M, Keshavarzian A, Kottapalli V, Badie B, Winship D, Fields JZ (1996). Human neutrophil functions are inhibited in vitro by clinically relevant ethanol concentrations.. Alcohol Clin Exp Res.

[25] Vasdev S, Gill V, Singal PK (2006). Beneficial effect of low ethanol intake on the cardiovascular system: possible biochemical mechanisms.. Vasc Health Risk Manag.

[26] Rehm J, Baliunas D, Borges GL, Graham K, Irving H, Kehoe T (2010). The relation between different dimensions of alcohol consumption and burden of disease: an overview.. Addiction.

[27] Jurkovich GJ, Rivara FP, Gurney JG, Fligner C, Ries R, Mueller BA (1993). The effect of acute alcohol intoxication and chronic alcohol abuse on outcome from trauma.. JAMA.

[28] Lustenberger T, Inaba K, Barmparas G, Talving P, Plurad D, Lam L (2011). Ethanol intoxication is associated with a lower incidence of admission coagulopathy in severe traumatic brain injury patients.. J Neurotrauma.

[29] Relja B, Menke J, Wagner N, Auner B, Voth M, Nau C (2016). Effects of positive blood alcohol concentration on outcome and systemic interleukin-6 in major trauma patients.. Injury.

[30] Wagner N, Akbarpour A, Mors K, Voth M, Stormann P, Auner B (2016). Alcohol intoxication reduces systemic interleukin-6 levels and leukocyte counts after severe TBI compared with not intoxicated TBI patients.. Shock.

[31] Taieb J, Delarche C, Ethuin F, Selloum S, Poynard T, Gougerot-Pocidalo MA (2002). Ethanol-induced inhibition of cytokine release and protein degranulation in human neutrophils.. J Leukoc Biol.

[32] Relja B, Omid N, Wagner N, Mors K, Werner I, Juengel E (2016). Ethanol, ethyl and sodium pyruvate decrease the inflammatory responses of human lung epithelial cells via Akt and NF-kappaB in vitro but have a low impact on hepatocellular cells.. Int J Mol Med.

[33] Pruett SB, Zheng Q, Fan R, Matthews K, Schwab C (2004). Ethanol suppresses cytokine responses induced through Toll-like receptors as well as innate resistance to Escherichia coli in a mouse model for binge drinking.. Alcohol.

[34] Relja B, Omid N, Kontradowitz K, Jurida K, Oppermann E, Stormann P (2014). Decreased inflammatory responses of human lung epithelial cells after ethanol exposure are mimicked by ethyl pyruvate.. Mediators Inflamm.

[35] Relja B, Henrich D, Wetzel G, Sander AL, Jakob H, Maraslioglu M (2013). Effects of acute ethanol gavage on intestinal integrity after hemorrhage/resuscitation.. Scand J Gastroenterol.

[36] Relja B, Wilhelm K, Wang M, Henrich D, Marzi I, Lehnert M (2012). Acute ethanol gavage attenuates hemorrhage/resuscitation-induced hepatic oxidative stress in rats.. Oxid Med Cell Longev.

[37] Hill DB, Marsano L, Cohen D, Allen J, Shedlofsky S, McClain CJ (1992). Increased plasma interleukin-6 concentrations in alcoholic hepatitis.. J Lab Clin Med.

[38] Lin HZ, Yang SQ, Zeldin G, Diehl AM (1998). Chronic ethanol consumption induces the production of tumor necrosis factor-alpha and related cytokines in liver and adipose tissue.. Alcohol Clin Exp Res.

[39] Goral J, Choudhry MA, Kovacs EJ (2004). Acute ethanol exposure inhibits macrophage IL-6 production: role of p38 and ERK1/2 MAPK.. J Leukoc Biol.

[40] MacGregor RR, Safford M, Shalit M (1988). Effect of ethanol on functions required for the delivery of neutrophils to sites of inflammation.. J Infect Dis.

[41] Jonsson AS, Palmblad JE (2001). Effects of ethanol on NF-kappaB activation, production of myeloid growth factors, and adhesive events in human endothelial cells.. J Infect Dis.

[42] Li X, Kovacs EJ, Schwacha MG, Chaudry IH, Choudhry MA (2007). Acute alcohol intoxication increases interleukin-18-mediated neutrophil infiltration and lung inflammation following burn injury in rats.. Am J Physiol Lung Cell Mol Physiol.

[43] Chen MM, O’Halloran EB, Shults JA, Kovacs EJ (2016). Kupffer cell p38 mitogen-activated protein kinase signaling drives postburn hepatic damage and pulmonary inflammation when alcohol intoxication precedes burn injury.. Crit Care Med.

[44] Qin Y, Hamilton JL, Bird MD, Chen MM, Ramirez L, Zahs A (2014). Adipose inflammation and macrophage infiltration after binge ethanol and burn injury.. Alcohol Clin Exp Res.

[45] Bergamini CM, Gambetti S, Dondi A, Cervellati C (2004). Oxygen, reactive oxygen species and tissue damage.. Curr Pharm Des.

[46] Kurose I, Higuchi H, Miura S, Saito H, Watanabe N, Hokari R (1997). Oxidative stress-mediated apoptosis of hepatocytes exposed to acute ethanol intoxication.. Hepatology.

[47] Wu D, Cederbaum AI (1999). Ethanol-induced apoptosis to stable HepG2 cell lines expressing human cytochrome P-4502E1.. Alcohol Clin Exp Res.

[48] Fu P, Peng C, Ding JY, Asmaro K, Sullivan JM, Guthikonda M (2013). Acute administration of ethanol reduces apoptosis following ischemic stroke in rats.. Neurosci Res.

[49] Wang F, Wang Y, Geng X, Asmaro K, Peng C, Sullivan JM (2012). Neuroprotective effect of acute ethanol administration in a rat with transient cerebral ischemia.. Stroke.

[50] Kany S, Woschek M, Kneip N, Sturm R, Kalbitz M, Hanschen M (2018). Simvastatin exerts anticancer effects in osteosarcoma cell lines via geranylgeranylation and c-Jun activation.. Int J Oncol.

